# Conserved and Variable Functions of the σ^E^ Stress Response in Related Genomes

**DOI:** 10.1371/journal.pbio.0040002

**Published:** 2005-12-20

**Authors:** Virgil A Rhodius, Won Chul Suh, Gen Nonaka, Joyce West, Carol A Gross

**Affiliations:** **1** Department of Microbiology and Immunology, University of California, San Francisco, California, United States of America; **2** Department of Cell and Tissue Biology, University of California, San Francisco, California, United States of America; The Institute for Genomic ResearchUnited States of America

## Abstract

Bacteria often cope with environmental stress by inducing alternative sigma (σ) factors, which direct RNA polymerase to specific promoters, thereby inducing a set of genes called a regulon to combat the stress. To understand the conserved and organism-specific functions of each σ, it is necessary to be able to predict their promoters, so that their regulons can be followed across species. However, the variability of promoter sequences and motif spacing makes their prediction difficult. We developed and validated an accurate promoter prediction model for *Escherichia coli* σ^E^, which enabled us to predict a total of 89 unique σ^E^-controlled transcription units in *E. coli* K-12 and eight related genomes. σ^E^ controls the envelope stress response in *E. coli* K-12. The portion of the regulon conserved across genomes is functionally coherent, ensuring the synthesis, assembly, and homeostasis of lipopolysaccharide and outer membrane porins, the key constituents of the outer membrane of Gram-negative bacteria. The larger variable portion is predicted to perform pathogenesis-associated functions, suggesting that σ^E^ provides organism-specific functions necessary for optimal host interaction. The success of our promoter prediction model for σ^E^ suggests that it will be applicable for the prediction of promoter elements for many alternative σ factors.

## Abstract 2

A model for predicting the variable promoter sequences associated with the bacterial stress response is developed and used to identify constituents of the transcriptional response to σ^E^.

## Introduction

Induction of alternative sigma (σ) factors is an important strategy for coping with environmental stress in bacteria. Indeed, there is a rough correlation between the apparent complexity of the environment and the number of alternative σ factors, e.g., *Mycoplasma* sp., which are obligate intracellular pathogens, contain only the housekeeping σ and no alternative σ's; *Escherichia coli,* which inhabits the relatively constant environment of its host organisms but can also survive in vitro, has six alternative σ's; and *Streptomyces coelicolor,* which inhabits a hostile and changing soil environment, has 62 alternative σ's. Therefore, the ability to predict promoters recognized by alternative σ's would significantly improve our capacity for understanding how bacteria adapt to stress.

It is challenging to predict bacterial promoters, which are composed of two conserved sequences centered at about −10 and −35 from the start point of transcription. Some promoters also have an “upstream element” (UP) upstream of the −35 sequence and/or an “extended −10” element immediately upstream of the −10. The fact that these promoters are composed of multiple, weakly conserved elements separated by less conserved, variable length spacer sequences makes their prediction a difficult bioinformatics problem. Such attempts have a long history, mostly directed at predicting promoters recognized by σ^70^ (b3067), the housekeeping σ in *E. coli,* using hidden Markov models, neural networks [1–4], and position weight matrices (PWMs) [5–8]. While these methods detect promoters with a moderate degree of success, they suffer from high false-positive rates (FPRs) in genomic sequences. In addition, promoter consensus and mismatch searches have also been employed to identify promoters for the Group IV factor, σ^W^ (Bsu0173), in *Bacillus subtilis* [9]. However, these approaches are not as effective as using PWMs that better describe the natural variability of target sites. Here, we consider only PWMs because their success is comparable to more complex models [3]. Staden [5] used three matrices (describing the −35, −10, and +1 promoter motifs) and one spacer penalty (for the −35 to −10) to predict σ^70^ promoters; variations of this approach were later explored by Hertz and Stormo [7]. Huerta and Collado-Vides describe the most accurate prediction method to date for σ^70^ promoters using multiple matrices for the −35 and −10 motifs, with one spacer penalty for the intervening spacer [6]. Although this method successfully identifies known promoters with high sensitivity (86%; true positives/total promoters), it suffers from many false predictions resulting in low precision (20%; true positives/total predictions), reducing its utility as a prediction tool to identify new promoters.

Alternative σ factors usually turn on a group of genes synchronously in response to a particular stress, and hence use very few activators. As a consequence, promoters recognized by alternative σ factors are somewhat less variable and might have higher information content than those recognized by the housekeeping σ factor, making them more amenable to bioinformatic analysis. We chose to test this proposition by determining the feasibility of predicting promoters of *E. coli* σ^E^ (b2573), both in *E. coli* K-12 and in related bacteria. σ^E^, a Group IV (extracytoplasmic, ECF) σ factor [10,11], mediates the envelope stress response [12,13], is essential in *E. coli* K-12 [14], and is important for virulence in related bacteria [15–22]. We first identified σ^E^ regulon members and their promoters using genome-wide expression analysis and transcript start site mapping in the *E. coli* K-12 genome. We derived a model for these σ^E^ promoters by building upon approaches pioneered for σ^70^ promoters, and used this model to make predictions in related genomes. By comparing promoter predictions from the actual genome with those from “randomized” genomes, we were able to identify those promoters that are unlikely to occur by chance alone. In addition, we adapted cross-genome approaches utilized for transcription factors [23–25] as an additional way of predicting promoters in *E. coli* and related pathogenic genomes. We tested all predictions in *E. coli* K-12 and *Salmonella typhimurium* and unique predictions in *E. coli* CFT073. These tests demonstrated that the model works with high precision.

Our studies reveal that the extended regulon of 89 predicted transcription units (TUs) is predicted to consist of a core set of genes conserved in most organisms and another group of more poorly conserved genes. Remarkably, each of these gene sets has a coherent function. The core genes coordinate the assembly and maintenance of lipopolysaccharide (LPS) and outer membrane porins (OMPs), the two key structures of the outer membrane of Gram-negative bacteria, in response to environmental change. A majority of the variable σ^E^ regulon members perform functions known to be important for a pathogenic lifestyle. We suggest that induction of such determinants at the first sign of stress facilitates bacterial adaptation to the host environment.

## Results

### Identifying σ^E^-Dependent Genes by Transcription Profiling

**Figure 1:**
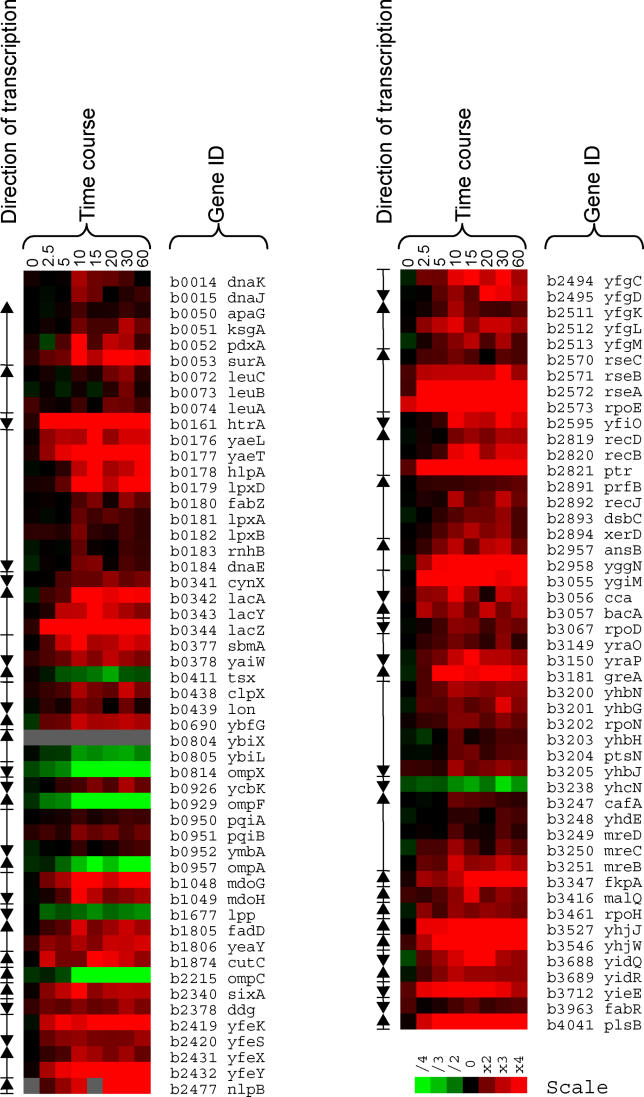
Expression Profiles of σ^E^ Regulon Members Significantly regulated genes identified from genome-wide transcription profiling following comparison of *rpoE* overexpressed (CAG25197) versus wild-type (CAG25196) *E. coli* K-12 MG1655 cells. The color chart illustrates the expression level for each gene from an average of four time-course experiments (see Materials and Methods). Red denotes induced, and green denotes repressed genes in CAG25197 following *rpoE* induction. Fold change of mRNA levels (*rpoE* overexpressed/wild-type) is indicated by the scale at the bottom of the figure; time in minutes after induction of *rpoE* in the time-course experiments is indicated at the top of the figure. Genes are identified by their unique ID and name (Gene ID) and are listed in chromosomal order to illustrate the TUs; the direction of transcription is indicated.

σ^E^-dependent genes were initially identified using genome-wide transcription profiling, comparing a wild-type *E. coli* K-12 strain that has a low level of σ^E^, with a strain overexpressing σ^E^ (following induction of its gene, *rpoE,* from an inducible promoter by IPTG). This strategy is preferable to comparison with an *rpoE^−^* strain because: (1) many σ^E^-transcribed genes have multiple promoters, so that the change in transcriptional signal upon loss of σ^E^ is often small; and (2) *rpoE^−^* strains (which require an uncharacterized suppressor for viability [14]) grow slowly, invalidating the direct comparison between *rpoE^+/−^* strains. We monitored changes in gene expression in four separate time-courses after induction and used statistical analysis of microarrays (SAM) [26] to identify 75 significantly induced and eight significantly repressed genes (Figure 1; see Materials and Methods). Some of these genes are part of operons in which other gene members were clearly induced but were not marked as significant in our strict selection criteria. Therefore, to fully describe the σ^E^ regulon we expanded this set by using the statistics from SAM to analyze the reproducibility and significance of the expression ratios of all the genes adjacent to and in the same orientation as the highly significant genes. This gave 96 genes organized in 50 σ^E^-dependent TUs, of which 42 were induced and eight were repressed (Figure 1).

### Identification of σ^E^ Promoter Motifs Upstream of Induced TUs

To determine which of our induced genes might have σ^E^ promoters, we used rapid amplification of cDNA ends (5′ RACE; see Materials and Methods) to identify start points of each TU, comparing mRNAs from *rpoE* overexpressed versus *rpoE^−^* cells. This analysis indicated that 28 of the 42 induced TUs contained σ^E^-dependent transcription start sites (unpublished data). The remaining promoterless TUs identified in transcriptional profiling may be indirectly regulated by σ^E^, especially since most were only weakly induced.

Bacterial promoters are located immediately upstream of their start sites. We therefore searched small blocks of sequences directly upstream of the 5′ RACE determined transcription starts for conserved σ^E^ motifs using the algorithm WCONSENSUS (see Materials and Methods). By testing several different search-window positions and widths, we found that a 16-nt search window (−1 to −16) was optimal for identifying the conserved −10 motif (T/ CGGTCAAAA), and that a 16-nt search window starting 9 nt upstream of the −10 element was optimal for locating the −35 motif ( GGAACTTTT). Although there were no other highly significant motifs, we found a 30-nt window of generally A/T-rich sequences directly upstream of the −35 motif with two conserved A/T-rich elements at positions −48/−49 and −57/−58. These correspond closely to the two information peaks in the SELEX-derived consensus sequences for the UP element of the *rrnB* P1 promoter [27]. In addition, the initiation nucleotide of the 28 promoters exhibited a strong preference for a purine (A/G) and weak conservation of sequences directly upstream.

**Figure 2:**
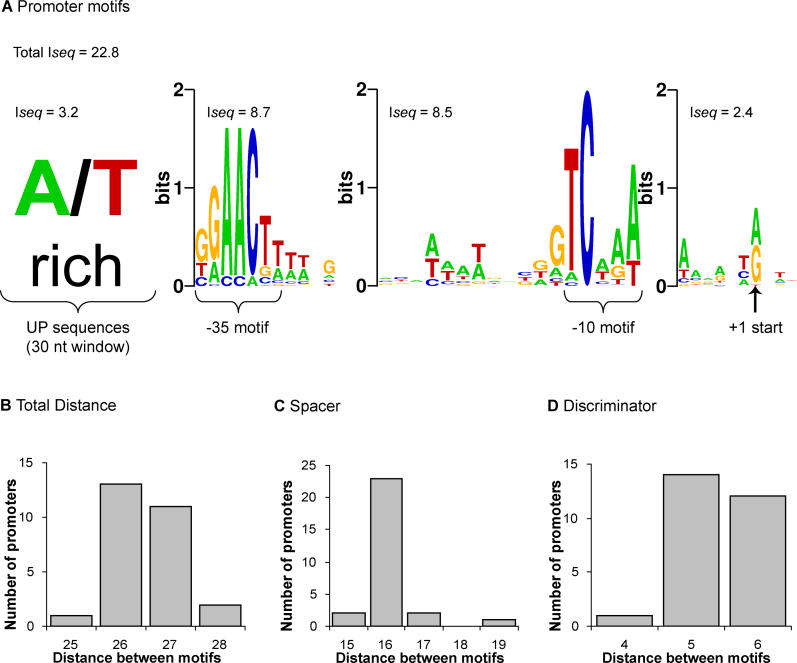
Sequence Logos and Spacer Histograms of σ^E^ Promoter Motifs Motifs were identified upstream of the 28 mapped transcription starts in *E. coli* K-12. (A) Sequence logos (http://weblogo.berkeley.edu/; [78]) of the −35, −10, and +1 start site motifs and the A/T rich UP sequences. The information content *(I_seq_)* of each motif is indicated (see Materials and Methods). (B–D) Histograms of the number of promoters versus distances between the motifs identified in (A): (B) +1 start and −35 motifs; (C) −10 and −35 motifs; and (D) +1 start and −10 motifs. Distances between the −35, −10, and +1 start motifs are from the conserved GGAACTT, TCAAA, and A/G sequences, respectively, as marked in (A). Note that the weakly conserved spacer sequence appeared to associate with the −10 motif and was therefore incorporated into PWM_−10_.

The sequence logos of the conserved sequence motifs upstream of the 28 σ^E^-dependent transcription start sites, together with their information content, are displayed in Figure 2A. The fact that all of the sequences contained good −35 and −10 promoter motifs indicated that we had successfully mapped σ^E^-dependent transcription initiation sites. Note that most of the total information content of the promoter motifs (22.8 bits) was contributed by the well-conserved −10 and −35 motifs. Figure 2B–2D displays histograms of the distance distributions of the promoter elements from each other: most promoters preferred a 5/6-nt discriminator region between the −10 and +1 (Figure 2D), while the spacing between the −10 and −35 varied from 15–19 nt, with 16 nt strongly preferred (Figure 2C). Interestingly, individual promoters displayed an inverse correlation between the length of these two spacers: promoters with a long −10/−35 spacer tended to have a short discriminator, and vice versa. Consequently, the range of distances between the −35 and +1 for all the promoters is quite small: 25–28 nt, with most promoters preferring a 26/27-nt spacer (Figure 2B). The identified promoter sequences are listed in section A of Table 1.

**Table 1:**
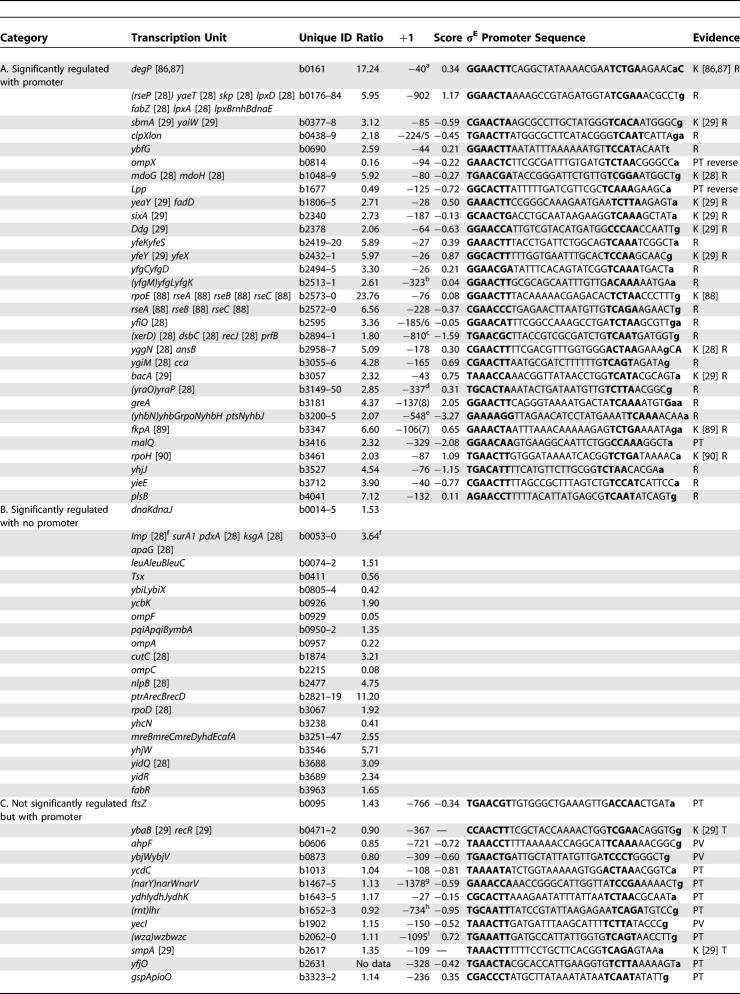
σ^E^ Regulon Members in *E. coli* K-12

**Table 1:**
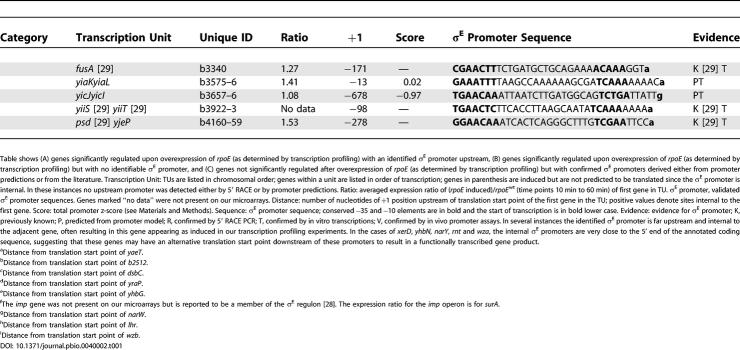
Continued

### Genome-Wide Predictions of σ^E^ Promoters

The sequence alignments for the UP, −35, −10, and +1 sequences were used to build four PWMs (see Materials and Methods); each PWM spans the complete sequence illustrated in each logo in Figure 2A. Each promoter was then scored by summing the individual PWM scores and incorporating penalties for suboptimal spacing between the motifs to generate a distribution of known promoter scores with mean (*μ_k_*) and standard deviation (*σ_k_*). High-scoring promoters were composed of more highly conserved promoter elements at optimal spacings, and low-scoring promoters contained less well-conserved elements at suboptimal spacings.

We searched the *E. coli* K-12 MG1655 sequence for σ^E^ promoters in which each individual PWM scored ≥μ−2σ, and where the distance between motifs was within the range observed for the 28 RACE-identified promoters. These constraints allow potential promoters to have a combination of weak and strong motifs and the variable spacings characteristic of known *E. coli* K-12 σ^E^ promoters. Genome-wide predictions with PWM_-35_ identified 98,113 sites (Table 2). Sequences flanking these sites were then searched for UP, −10, and +1 motifs within the spacing range of our validated promoters to create a library of candidate promoters (note that the order of the searches does not affect the final library). The total promoter score of each candidate was calculated using the same procedure described above for the known promoters and then converted to a z-score (the number of standard deviations [*σ_k_*] of the candidate score from the mean score of the known promoters [*μ_k_*]). In cases where promoters overlapped such that the +1 motifs were within 4 nt of each other, only the highest scoring promoter was selected. This generated a library of 553 candidate promoters that includes 27 of the 28 RACE-identified promoters (Table 2), missing only the *ybfG* promoter that fails due to a poor start motif (<μ−2σ) despite having a relatively high total promoter z-score (−0.03).

**Table 2:**
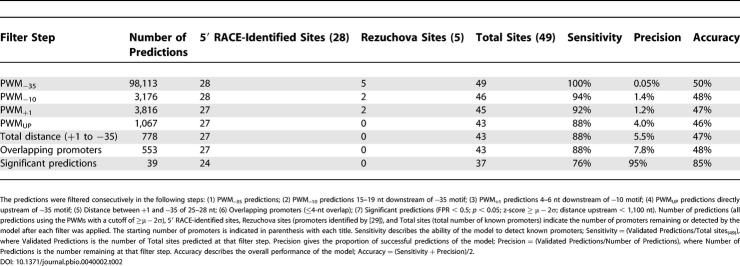
Genome-Wide σ^E^ Promoter Predictions in *E. coli* K-12

### Identifying Significant σ^E^ Promoters From the Promoter Prediction Library

**Figure 3:**
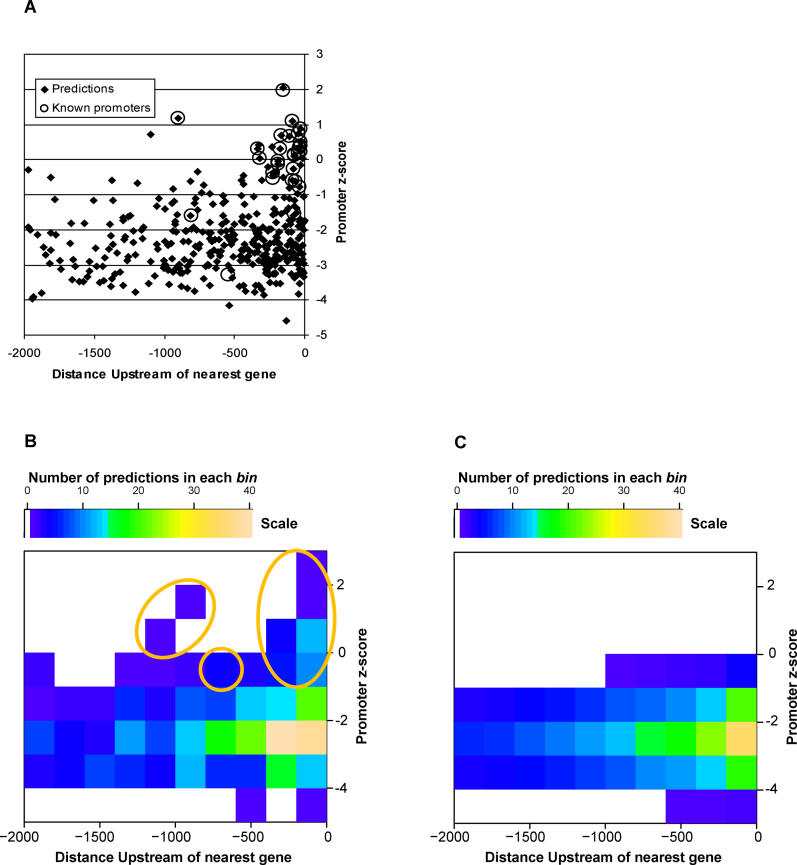
σ^E^ Promoter z-Scores versus Distance Upstream of the Nearest Gene in Actual and Randomized *E. coli* K-12 Genomes Only promoters less than 2,000 nt upstream of target genes are shown. (A) Scatter plot of predicted (diamonds) and known (circles) σ^E^ promoters in *E. coli* K-12 MG1655. (B) Topographic plot of predicted σ^E^ promoters in *E. coli* K-12 MG1655. The *x* and *y* axes are divided up into 200-nt and 1 unit *bins*, respectively, and the number of predictions falling within each *bin* are indicated colorimetrically as shown in the scale. Note that the data in this plot are the same as the predictions in (A). *Bins* containing significant predictions are indicated by yellow ovals. (C) Topographic plot indicating average number of predicted σ^E^ promoters made from 100 randomized *E. coli* K-12 MG1655 genomes in silico (see Materials and Methods). Each *bin* illustrates the average number of predictions made from 100 separate randomized genomes that fall within the parameters of that *bin*.

The vast majority of the 553 predicted promoters were low scoring and randomly distributed, in contrast to the 5′ RACE validated promoters, which were high scoring (> −1) and located near target genes (Figure 3A). To identify significant (i.e., functional) promoters from our library, we compared predictions from the actual genomic sequence (Figure 3B) with those from 100 randomized genomes generated in silico (Figure 3C). The randomized genomes maintain the location of all open reading frames (ORFs), average codon, and nucleotide content, but now contain only nonspecific sequences. Hence, predictions from these genomes indicate the number of predictions occurring by chance alone. This allows us to determine both a FPR and a probability score that the prediction arose by chance (*p*-value) for every prediction in the actual K-12 genome. Using a cutoff of FPR <0.5 and *p* < 0.05 for each *bin* (a *bin* describes a group of promoters with similar scores and positions relative to the gene) and an additional distance and z-score constraint to remove spurious predictions (see Materials and Methods), we generated 39 highly significant predictions. Their combined FPR is 0.22, which means that 8.6 of 39 predictions would be expected by chance alone. Of the 39 significant predictions, 24 were of previously validated promoters located upstream of genes that were induced in transcriptional profiling. The remaining 15 predicted promoters were not upstream of genes that were induced in transcriptional profiling. Interestingly, one promoter is upstream of *ompX* (b0814), which is repressed in the transcription profiling, but is oriented away from the gene. Thirteen of 15 promoters (including *ompX*) were confirmed either by in vitro transcription or in vivo promoter assays (sections A and C in Table 1), giving a total of 37 of 39 verified significant predictions.

### How Well Does Our σ^E^ Promoter Model Perform in *E. coli* K-12?

To determine the performance of our model in identifying significant promoters, we need to know the total number of validated σ^E^ promoters in *E. coli* K-12. We used several approaches to identify the 49 promoters that comprise the σ^E^ regulon in this organism (all promoters are listed in Table 1). (1) We identified 28 promoters by transcriptional profiling coupled with 5′ RACE and 13 additional promoters from our significant promoter model to give 41 promoters. (2) We searched our library of 553 promoters for any new predictions upstream of genes that were induced in our transcriptional profiling experiments. We found two low-scoring promoters located upstream of genes (*malQ* [b3416] and *lpp* [b1677]); these were validated in vitro to give 43 promoters. Note that, similar to *ompX, lpp* is repressed in the transcription profiling and the σ^E^ promoter is upstream but oriented away from the gene. (3) We noticed that several validated predictions are located far upstream of the nearest gene (*dsbC* [b2893], *yhbG* [b3201], *lhr* [b1653], and *wzb* [b2061]; Table 1) and are in fact internal and very close to the 5′ end of the adjacent ORF, suggesting that these ORFs may be misannotated. Searching our promoter library, we found a high-scoring promoter located upstream of *narW* (b1466) just beyond our distance cut-off that was very close to the beginning of *narY* (b1467). We confirmed this promoter in vitro to give 44 promoters. (4) Two genetic screens [28,29] identified additional putative σ^E^- dependent promoters; we validated the five additional promoters identified by Rezuchova et al. to give 49 validated promoters, but were unable to validate any of the eight new promoters proposed by Dartigalongue et al. We note that most of the Dartigalongue et al.–proposed promoters contain poorly conserved sequence elements separated by a wide range of spacer lengths, suggesting they might not be functional. Table 3 shows all validated *E. coli* K-12 σ^E^ regulon members divided into functional categories.

**Table 3:**
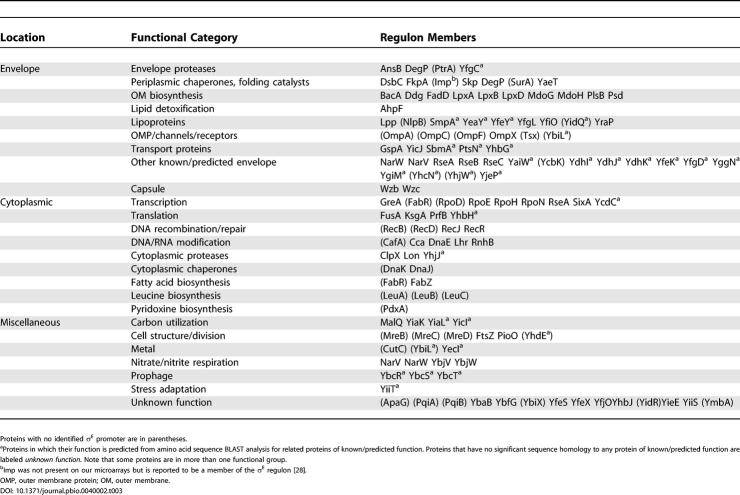
Functional Classification of the σ^E^ Regulon Members in *E. coli* K-12

**Figure 4:**
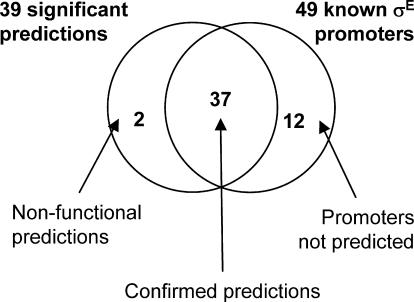
Venn Diagram of Predicted and Known σ^E^ Promoters in *E. coli* K-12 39 predictions from the promoter library were identified as highly significant, of which 37 were confirmed. A total of 49 known σ^E^ promoters were confirmed from the literature and additional experiments, of which 37 were successfully identified by the promoter prediction model (see text; Table 2).

Of the 39 highly significant predictions, 37 were validated, giving our promoter model a precision of 95% (validated predictions/number of predictions; Table 2 and Figure 4). This promoter model also successfully identified 37 of 49 known σ^E^ promoters, giving a sensitivity of 76% (validated predictions/known promoters). Averaging the sensitivity and precision scores gives an estimate of the total performance, or accuracy, of the σ^E^ prediction model (85%; Table 2). True promoters that remained undetected by the highly significant prediction model did so for a variety of reasons: five promoters failed because either their UP, −35, −10, or +1 motifs scored less than μ − 2σ; five promoters failed because of low total promoter scores, making them difficult to distinguish from the many other low-scoring nonfunctional promoters; and two failed because they were located far upstream of the nearest gene. Given the variety of reasons that they failed, this suggests that they were outliers rather than a fault with a particular predictive step of the model.

### Predictions of σ^E^ Promoters in Closely Related Genomes

Given the success of our promoter model in *E. coli* K-12, we extended it to eight genomes of closely related organisms in which the DNA binding determinants of the σ^E^ orthologs are identical or very similar to those in *E. coli* K-12 σ^E^ (Figure S1). This determination is based on the demonstration that the structure of Domain 2 (which recognizes the −10 conserved promoter sequence) and of Domain 4 (which recognizes the −35 conserved promoter sequence) of *E. coli* σ^E^ can be overlaid with that of σ^70^, the housekeeping σ, indicating that the structure of these two domains is conserved across σ's [30]. The −10 and −35 promoter recognition determinants in σ^70^ have been thoroughly mapped [31]. We assumed that comparable residues in σ^E^ carried out −10 and −35 recognition and identified eight organisms in which these residues were highly conserved.

We applied the promoter prediction model developed in *E. coli* K-12 to these eight genomes to generate a library of promoter predictions for each organism. We then identified all putative regulon members in TUs by assuming that the downstream genes formed an operon if they were in the same orientation and the intervening intergenic region (IG) was less than 50 nt [32]. Significant promoters were identified as described above for *E. coli* K-12 by comparison to predictions from random genomes (constructed specifically for each real genome to account for their structure, average codon, and nucleotide contents). To prevent spurious results in some genomes, significant promoters (FPR < 0.5; *p* < 0.05) were also filtered for z-score > −2 and distance < 1,100 nt upstream of genes.

As a second method, a significant prediction in any one genome was used to search the relevant promoter library for promoters upstream of conserved orthologs in the other species (see Materials and Methods). The matching promoter did not have to satisfy a minimum *p*-value or FPR, enabling the detection of less well-conserved orthologous promoters. However, to prevent spurious results, predicted σ^E^ promoters were required to have a z-score > −2 and to be within 1,100 nt upstream of the orthologous gene or TU. For each significant prediction upstream of a conserved ortholog, the probability of identifying a matching promoter in each genome by random chance from the promoter libraries is approximately 0.03, suggesting that the matches we identified were highly significant. In addition, we found that the vast majority of matching promoters were at similar distances upstream of the orthologs as the original search promoter, further increasing the significance of the matches. The results of these procedures are summarized in Table 4 and are presented in a database of conserved predicted σ^E^ promoters and regulon members across all nine genomes (Table S1).

**Table 4:**
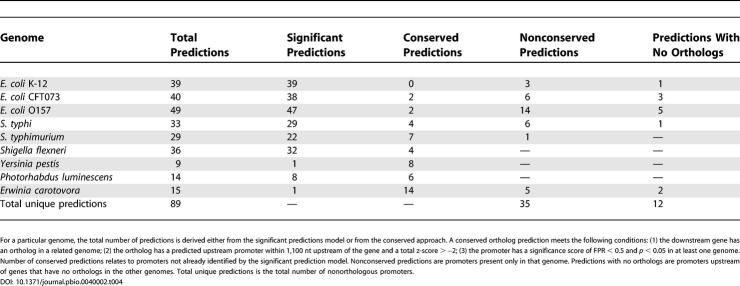
Genome-Wide σ^E^ Promoter Predictions in Nine Related Genomes

These two computational approaches, together with experimentally identified promoters in *E. coli* K-12, generated an “extended σ^E^ regulon” across nine genomes, which consisted of 89 unique TUs (Table 4). Interestingly, there are no TUs predicted to be regulated by σ^E^ in all nine genomes; however, a core of 19 TUs is present in at least six genomes. The conserved members of the regulon predominantly carry out related functions (Table 5) involving the outer membrane and the regulatory strategy to maintain the σ^E^ response. The majority of the remaining σ^E^-controlled TUs are not highly conserved, but most control cell envelope functions (Table 5; see Table S2 for a list of all the extended regulon members in each functional category).

**Table 5:**
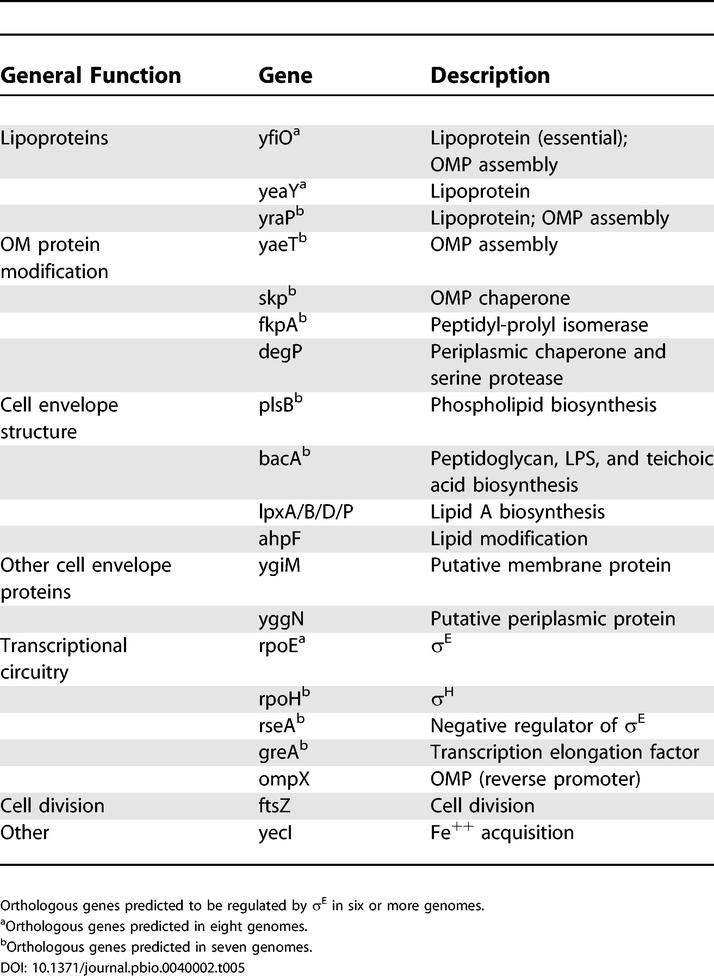
Predicted Core σ^E^ Regulon Members

Among the nine organisms, *E. coli* O157:H7 has the most predictions (49) and *Yersinia pestis* the least (nine) (Table 4). Genomes may have fewer significant σ^E^ predictions because they have a reduced σ^E^ regulon. Alternatively, the promoter model may not perform well in that organism. We believe that *Yersinia* is an example of an organism with a reduced σ^E^ regulon, making it difficult to detect its promoters with the random genome approach that relies on identifying overrepresented sequences. In support of this idea, the σ^E^ DNA–binding determinants in both organisms are essentially conserved (see Figure S1), and eight of nine *Yersinia* promoters with reasonable promoter scores were identified using the conserved ortholog approach (see Table 4). This may also be true for *Erwinia* and *Photorhabdus,* which also have only a few significant promoter predictions (one and eight, respectively). However, they also contain four and six amino acid changes, respectively, near the DNA-binding determinants of regions 2.4 and 4 (see Figure S1), so there is a possibility that there is a slight deviation of the optimal promoter sequence that is not captured by the *E. coli* promoter prediction model. We note, though, that these genomes still share many highly conserved σ^E^ regulon members, indicating that many of our predictions in these genomes should be functional. In more divergent genomes, where σ^E^ orthologs had amino acid changes at critical DNA-binding positions (*Shewanella oneidensis, Vibrio cholerae,* and *Pseudomonas aeruginosa;* unpublished data), our model was unsuccessful. Interestingly, loss of *P. aeruginosa* σ^E^ is complemented by *E. coli* σ^E^ [21], and likewise, both σ^E^ consensus sequences are similar ([33] and references therein). However, few promoters match consensus, and the σ^E^ orthologs may tolerate different variations in their target promoter sequences.

### Validation of the σ^E^ Promoter Model in *S. typhimurium* and *E. coli* CFT073

To determine the validity of our predictions, we experimentally tested all predictions made in *S. typhimurium.* In addition, we tested all unique predictions made in *E. coli* CFT073 (conserved predictions were not tested because their promoters were virtually identical to those found in *E. coli* K-12). Promoter function was tested both by in vivo promoter assay (see Materials and Methods) and in vitro transcription (see Tables S1 and S2). Although both of these assays used *E. coli* K-12 RNA polymerase and σ^E^, we do not think there are any functional differences from the *E. coli* CFT073 and *S. typhimurium* σ^E^ holoenzymes since their subunits are virtually identical and differ only in a few nonessential positions, with at least 99.72% and 98.58% sequence identity, respectively, with the *E. coli* K-12 subunits. These assays revealed a high success rate. For *S. typhimurium,* we made a total of 29 predictions, composed of 22 significant predictions based on the random genome model and seven predictions based on the conserved ortholog approach. Sixteen of 22 (73%) of the significant predictions and four of seven (59%) of the conserved orthologs were validated, for an overall success rate of 69%. For CFT073, of the 40 predictions, we have validated 29 of 38 (76%) significant predictions and two of two conserved ortholog predictions, for an overall success rate of 78%. We note that unconfirmed predictions may still be functional in vivo, as they might require a coregulator not present in our assay conditions or in *E. coli* K-12. These results suggest that our promoter prediction strategies provide a reasonably accurate picture of the σ^E^ regulon in organisms closely related to *E. coli* K-12.

## Discussion

The goal of this work was to follow the responses mediated by alternative σ's across organisms to determine whether these responses have changed. This required us to develop methods that accurately predict promoters recognized by alternative σ's. We have developed a successful strategy to predict the σ^E^ regulon in *E. coli* K-12 and related organisms and have validated predictions in three organisms. We report the first comprehensive analysis of the conservation and variation of a σ factor regulon across genomes, identifying an “extended” σ^E^ regulon in nine genomes comprised of 89 unique TUs. Of these, only 19 are highly conserved. The highly conserved TUs maintain appropriate cellular levels of LPS and OMPs, two unique constituents of the outer membrane of Gram-negative bacteria, thereby identifying the core function of the regulon. The less-conserved regulon members perform multiple pathogenesis-associated functions, suggesting that the σ^E^ regulon has been co-opted to provide organism-specific functions necessary for optimal interaction with the host.

### Promoter Predictions

We chose to employ de novo promoter prediction as our primary method for cross-genome analysis because it can identify promoters unique to a particular genome. This is an important attribute, given the variability of bacterial genomes. For example, the three sequenced *E. coli* genomes share only 40% of their coding sequence. As a secondary approach, we searched for weakly conserved predictions upstream of orthologous genes, thereby identifying additional promoters too weak to pass the first filter (e.g., the latter method identified seven new *S. typhimurium* promoters, four of which were validated in vitro, and eight new *Yersinia* promoters). Our σ^E^ promoter model performed considerably better (precision = 95%; accuracy = 85%; see Table 2) than the housekeeping σ^70^ promoter model (precision = 20%) upon which it is based [5–7], primarily because the combined information content for σ^E^ is much higher than that for σ^70^ (*I*_seq_ = 22.8 bits versus 12.56 bits). In addition, performance was improved by comparison to a random genome to reduce false positives and our secondary approach of searching for conserved orthologs. Interestingly, σ^70^ promoters, but not σ^E^ promoters, were often embedded in predicted clusters of overlapping sites [6]. This distinction may result from the differences in specificity of the two models or reflect a fundamental distinction in promoter recognition mechanisms of housekeeping and alternative σ's. We note that a simple prediction model having a single-weight matrix and a fixed-length spacer suffices to predict promoters of another family of σ factors (σ^54^; RpoN) unrelated to the σ^70^ family [34–36]. In contrast, our promoter prediction model should be applicable for the prediction of promoters elements for the many alternative σ^70^ family members that bind to promoter elements separated by variable spacers, and especially Group IV σ's that tend to bind to more highly conserved promoter sequences [11].

Many σ^E^ promoter predictions were limited to particular subgroups. In some cases, the orthologs themselves had limited distribution. This particularly interesting case suggests that the ortholog has an organism or species-specific role. For example, the highly related *E. coli* and *Shigella* genomes contained three predictions upstream of orthologs exclusive to at least three of four of these genomes, and the two *Salmonella* species contained two predictions upstream of orthologs unique to *Salmonella* (see Table S1). In other cases, the orthologs themselves were widely distributed, but σ^E^ promoters were identified for only some orthologs. For example, ten predicted σ^E^ promoters are found only upstream of genes in *E. coli* and *Shigella,* and five σ^E^ promoters are found only upstream of genes in *Salmonella* (see Table S1). These cases may identify examples of regulon evolution, where σ^E^ promoters are created or lost in response to the requirements of the organism. Alternatively, we may have failed to detect σ^E^ promoters because one or more of their motifs failed our cutoff criteria. Finally, when σ^E^ promoters regulate long polycistronic TUs, some downstream TUs may no longer be classified as σ^E^ regulated in related genomes, either because of gene shuffling or because their intergenic distance was >50 nt (our cut-off for genes in an operon). In this latter case, σ^E^ might still regulate the downstream genes.

### The Core σ^E^ Regulon

**Figure 5:**
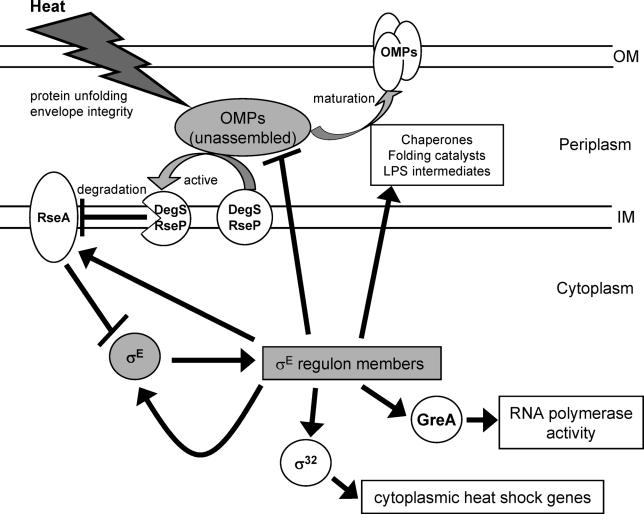
Functions of the Highly Conserved σ^E^ Core Regulon Members Stresses such as heat lead to the accumulation of unassembled OMPs; this activates the sequential proteolysis of the membrane-spanning antisigma RseA [12,54]. The inner membrane proteases DegS [b3235] and RseP [b0176] release the cytoplasmic portion of RseA, which is then degraded by the cytoplasmic proteases ClpX [b0438] and Lon [b0439] ([85]; R. Chaba unpublished data) to release free σ^E^, which then binds to RNA polymerase core to regulate the expression of target regulon members. σ^E^ up-regulates functions required for synthesis, assembly, and/or insertion of both OMPs and LPS, the most abundant components of the outer membrane, as well as envelope-folding catalysts and chaperones. σ^E^ also up-regulates expression of itself and its negative regulator RseA and enhances expression of GreA [b3181] and σ^32^ [b3461]. Importantly, σ^E^ down-regulates OMP expression, thereby reducing the accumulation of unassembled OMPs, which presumably limits the duration of the response.

The core σ^E^ regulon consists of 19 TUs and 23 proteins, of which 20 have known functions (Table 5; Figure 5). Amazingly, at least 60% of the core regulon members (~75% of proteins with known functions) ensure the synthesis and assembly of LPS and OMPs, or encode the transcriptional circuitry to maintain the homeostasis of these two key constituents of the outer membrane of Gram-negative bacteria. The proper ratio of OMPs and lipid A contributes to the impermeability of the outer membrane [37].

Five members of the core regulon are involved in the synthesis or assembly of LPS. Four members (Lpx A, B, D, and PlsB) promote the synthesis of lipid A, the hydrophobic anchor of the LPS, and a fifth (BacA) contributes to LPS assembly [38,39]. Lipid A comprises the outer leaflet of the outer membrane. The high resistance of Gram-negative bacteria to hydrophobic compounds is in large part due to the high density of saturated fatty-acid chains and potential for many lateral interactions in lipid A, which together dramatically slow diffusion of hydrophobic compounds through the outer membrane [40].

OMPs are trimeric β-barrel proteins that form channels in the outer membrane to permit access of small solutes. These abundant proteins comprise about 25% of the surface area of the bacteria [37] and have a complex assembly pathway. Six members of the core regulon promote the OMP assembly: two lipoproteins (YfiO and YraP) [41,42], three chaperones (Skp, FkpA, and DegP) [41,43], and YaeT (Omp85), which is generally implicated in insertion of β-barrel proteins into the outer membrane of many species [44–46] and may also do so in *E. coli* [45,46]. YaeT functions in a complex with three lipoproteins (YfiO, YfgL, and NlpB) [42], of which only YfiO is in the core regulon. However, the other two lipoproteins may also turn out to be part of the conserved regulon as YfgL is predicted to be driven by a σ^E^ promoter in five organisms and, at least in K-12, NlpB (b2477) is induced by overexpression of σ^E^ through an unknown mechanism. The complex assembly pathways of LPS and porins are not completely known, but it is clear that the two are mutually dependent [47–52]. Thus, some conserved regulon members may actually function in both assembly pathways.

Intriguingly, FtsZ, a member of the core regulon, is involved in initiating cell division (reviewed in [53]). This raises the possibility that the σ^E^ regulon may be needed to synthesize the excess outer-membrane components required at the time of septation. Thus, its primordial function may have been to facilitate passage through the cell cycle. However, as these core components are essential for the integrity of the outer membrane, this response could easily be used as a primary defense mechanism to protect the barrier function of the cell in the face of environmental stress.

The core regulon also encodes the transcriptional circuitry that allows the cell to detect and respond to imbalances in LPS and OMPs to maintain envelope homeostasis. Unassembled OMPs activate the proteolytic cascade that degrades RseA (b2572) [54], the membrane-spanning antisigma factor that inhibits σ^E^ function (reviewed in [12]). As LPS intermediates participate in OMP assembly [47–52], the unassembled OMP signal reports on the status of both LPS and OMP maturation [55–60]. Two notable features of the transcriptional circuit encoded by the core regulon ensure a rapid and sensitive response to imbalances in OMP assembly. First, the *rpoErseABC* operon has two highly conserved σ^E^ promoters, one upstream of the entire operon and the second upstream of *rseA* (see Table S1). As a consequence of this arrangement, σ^E^ positively autoregulates itself, thereby ensuring a rapid increase in proteins required for OMP/LPS homeostasis, and up-regulates RseA to set up a negative feedback loop (Table 5; Figure 5). The fact that RseA synthesis is driven from two promoters is likely to dampen the response, reduce oscillation, and provide a sufficient excess of RseA to ensure rapid down-regulation following a decrease in unassembled OMPs. A second important feature of the response is a homeostatic loop that prevents further buildup of unassembled OMPs (Figure 5). At least in *E. coli* K-12, OmpA (b0957), OmpC (b2215), OmpF (b0929), and OmpX are down-regulated upon induction of σ^E^, thereby decreasing the flow of OMPs to the envelope. Down-regulation may be accomplished by production of σ^E^-regulated antisense small RNAs transcribed divergently from their negatively regulated OMPs (V. Rhodius, unpublished data). Intriguingly, the σ^E^ promoter divergent from *ompX* is a member of the core regulon (see Table S1 and Table 5), raising the possibility that OMP down-regulation is a conserved feature of the response.

### The Extended σ^E^ Regulon

More than 60 of the unique σ^E^-controlled TUs we have predicted are present in fewer than six of the nine genomes we have scanned; many are present in only a small subset of these genomes (see Table S1 and Table 4). However, the majority of those with known functions carry out a coherent theme: adaptation of the organism to the conditions encountered when the bacterium interacts with its eukaryotic host (Table S2; Table 6). This idea is presaged by two functions in the core regulon: an iron acquisition system (YecI) to facilitate growth in the iron-deficient host environment and a component of alkyl reductase (AhpF) to detoxify lipid hydroperoxides that may be generated during exposure to macrophages.

**Table 6:**
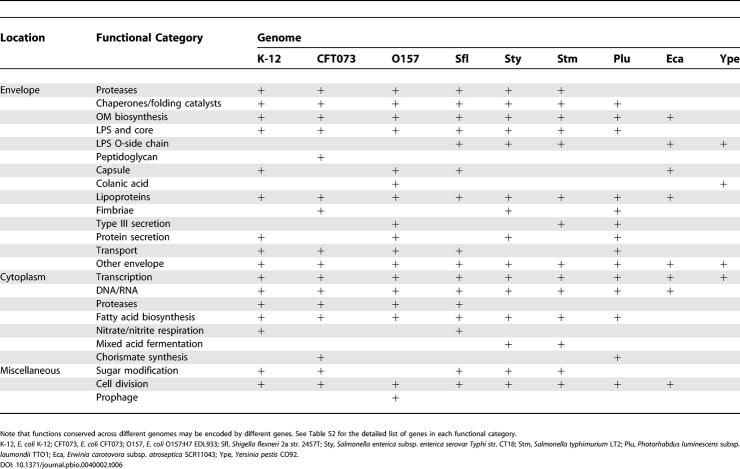
Predicted Properties of σ^E^ Regulon Members across Nine Genomes

The predicted extended regulon encodes multiple functions related to pathogenesis. Among these, several have already been validated in at least one organism. These include synthesis of capsule, a viscous polysaccharide layer that facilitates adhesion and protects against macrophage ingestion; recombination functions to resolve DNA lesions that could be generated by the respiratory burst (RecJ/O/R); and metabolic components for nitrate/nitrite respiration (NarW/V) that facilitate adaptation to the anaerobic/microaerophilic host environment. In addition, the regulon is predicted to encode components that produce colanic acid and chorismate and that modify the core and O-antigen portion of LPS, although no predictions in these classes have yet been validated. That the extended σ^E^ regulon encodes many pathogenesis-related functions explains why cells lacking σ^E^ are defective in pathogenesis [15–22], and suggests that the extended σ^E^ regulon may serve as an early adaptation system to facilitate survival in vivo. In addition, although the bacteria discussed here occupy diverse hosts, many pathogenic determinants apply broadly, even across the plant–animal divide [61–63].

Why is a response devoted to monitoring the status of OMPs and LPS also used for pathogenesis-related functions? Possibly, interaction with host cells alters the status of these σ^E^ regulators, thereby triggering the σ^E^ response. Using the core regulon as a base, organisms might then add additional members to the σ^E^ regulon that improve their viability in their hosts. This would explain why many of the pathogenesis functions are unrelated either to the core function of the regulon or even to the envelope itself. The variability of the σ^E^ regulon suggests that it may be easier to adapt the function of an existing regulator by changing the location of its binding sites than to evolve new regulators. Because environmental change is likely to generate envelope stress, it may be generally true that regulators sensing the envelope will contain organism-specific regulon members that facilitate the response for the particular ecological niche of the bacterium. Interestingly, σ^E^ is a member of the Group IV σ family, many of which also respond to stress in the envelope. It will be interesting to determine whether organism-specific variation in regulon function is characteristic of other Group IV σ's.

## Materials and Methods

### Media, strains, and plasmids

M9 complete minimal media was prepared as described [64], supplemented with 0.2% glucose, 1 mM MgSO_4_, vitamins, and all amino acids (40 μg/ml). The media was supplemented with 100 μg/ml ampicillin, 10 μg/ml tetracycline, and/or 20 μg/ml chloroamphenicol as required.

Bacterial strains and plasmids used in this study are listed in Table 7. Strain CAG25195 was constructed by using a lambda lysate from CAG16037 (MC1061 [Φλ*rpoH* P3::*lacZ*] Δ*lacX74*) to lysogenize MG1655 as described by [65]. P1 *vir*-mediated transductions were carried out as described by [66].

**Table 7:**
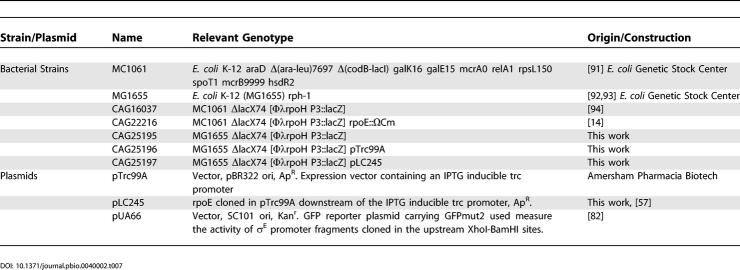
Bacterial Strains and Plasmids Used in This Study

Plasmid pLC245 was used to overexpress *rpoE* from the strong IPTG-inducible *trc* promoter and was constructed as follows: the *rpoE* gene was amplified by PCR from genomic MG1655 DNA using the primers RPOE1 (5′- CATATGAGCGAGCAGTTAACGGAC-3′) and RPOE2 (5′- GCAAGGATCCTCAACGCCTGATAAGCGGTT-3′), which encodes a BamHI site (underlined). The PCR product was digested with BamHI to create one overlapping end, and then ligated into vector DNA prepared from p*Trc*99A by digesting with EcoRI, treating with Klenow enzyme to produce a blunt end, and then digesting the vector with BamHI. The final construct was confirmed by sequencing.

### Strain growth and probe preparation for microarray analysis

To identify genes that alter their expression upon overexpressing σ^E^, time-course microarray experiments were performed with the strain CAG25196 (MG1655 Δ*lacX74* [Φλ*rpoH* P3::*lacZ*]) carrying the control vector, p*Trc*99A, versus CAG25197, which carries the IPTG-inducible *rpoE* overexpression vector, pLC245 (Table 7). Samples containing the control vector were labeled with Cy3 (green), and *rpoE* overexpression samples were labeled with Cy5 (red). Cells were grown in M9 complete minimal media with appropriate antibiotics in order to maximize the number of genes expressed, rather than in a rich media such as LB (luria broth) [67]. 500-ml conical flasks containing 100 ml of media were inoculated from fresh overnight cultures to a final OD_450_ = 0.03 or 0.035 for strains carrying the plasmid p*Trc*99A due to the fractionally slower growth rate. Cultures were grown aerobically at 30 °C in a gyratory water bath (model G76 from New Brunswick Scientific, Edison, New Jersey, United States) shaking at 240 rpm until OD_450_ = 0.3. Cultures were then induced with a final concentration of 1 mM IPTG and incubation resumed as before. Immediately prior to induction, and at 2.5, 5, 10, 15, 20, 30, and 60 min after induction, 1-ml and 8-ml samples were removed for microarray analysis.

Culture samples for microarray analysis were added to ice-cold 5% water-saturated phenol in ethanol solution, centrifuged at 6,600 *g,* and the cell pellets flash-frozen in liquid N_2_ before storing at −80 °C until required. Labeled probe for microarray analysis was prepared as described in [68]. Briefly, total RNA was isolated from the stored cell pellets using the hot phenol method, and labeled Cy3 and Cy5 cDNA was prepared from 16 μg of total RNA with 10 μg of random hexamer (Integrated DNA Technologies, Coralville, Iowa, United States) using the indirect labeling method.

### DNA microarray procedures

Relative mRNA levels were determined by parallel two-color hybridization to glass slide cDNA microarrays [69]. PCR products of 4,110 ORFs representing 95.8% of *E. coli* ORFs were prepared according to [70] using primers from SigmaGenosys (The Woodlands, Texas, United States). The products were spotted onto glass slides to make DNA arrays as described in protocols on http://derisilab.ucsf.edu/core/resources/index.html. Samples were hybridized to the arrays and scanned as described in [68]. The resulting TIFF images were analyzed using GenePix 3.0 software (Axon Instruments, Union City, California, United States) and the data stored on an AMAD database (software available from http://derisilab.ucsf.edu/core/resources/index.html).

### Expression data analysis

Expression data were normalized using the assumption that the quantity of initial mRNA was the same for both samples [71]. To correct for intensity (dye)–dependent biases, we used intensity-dependent normalization [72,73]. For each gene spot on an array, the green (Cy3) fluorescent intensity was defined as *G* = (F532_Median_ – B532) and the red (Cy5) fluorescent intensity was defined as *R* = (F635_Median_ – B635), where the local background intensity (B532, B635) is subtracted from the median foreground intensity (F532_Median_, F635_Median_). The data were filtered to exclude all *R* and *G* values less than 3 × local background. For each microarray experiment, an “MA-plot” was used to represent the *(R,G)* data, where *M* = log_2_ *R/G* and 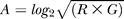 . A local *A*-dependent normalization was performed by fitting a normalization curve using the robust scatter plot smoother “lowess” implemented in the statistical software package R, such that:



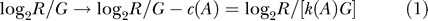



where *c*(*A*) is the lowess fit to the MA-plot. The fraction of data used for smoothing each point was 50%.

Statistically significant differentially expressed genes were identified from replicate microarray experiments using the SAM software ([26]; http://www-stat.stanford.edu/~tibs/SAM/index.html). SAM employs gene-specific *t* tests and by analyzing permutations of the *t* scores from the dataset derives a false discovery rate (percentage of genes identified by chance) for a user-selected cutoff threshold (the lowest false discovery rate at the median percentile). The *rpoE* time-course expression data revealed that genes that altered their expression in response to *rpoE* did so within 10 min after induction. Therefore, in each of the four time-courses time points from 10 min onwards were considered replicates and averaged to create four independent datasets. These data were then filtered for presence in at least 75% of datasets and significant genes identified using a stringent cutoff of the lowest false discovery rate (0.95%) at the median percentile.

### 5′ RACE PCR

The 5′ ends of σ^E^-dependent transcripts were mapped using new 5′ RACE adapted from [74]. We chose this method because (1) it is highly sensitive, facilitating the detection of weakly expressed transcripts; and (2) sequencing the RACE products enables the precise identification of mRNA 5′ ends. Total RNA was extracted as described for microarray analysis from strains CAG25197 (*rpoE* ^+^; Table 7) 1 h after induction with 1 mM IPTG and CAG22216 (*rpoE* ^−^; Table 7). Both strains were grown under identical conditions as for the microarray experiments in M9 complete minimal media with appropriate antibiotics to OD_450_ = 0.3; samples from CAG22216 were harvested, while CAG25197 was induced with 1 mM IPTG for 1 h before harvesting. Fourteen micrograms of total RNA was treated with 5 U tobacco acid pyrophosphatase (TAP; Epicentre Technologies, Madison, Wisconsin, United States) to remove the 5′ γ and β phosphates from the RNA, and the samples cleaned by organic extraction and ethanol precipitation. One hundred picomoles RNA oligo (5′- GAGGACU CGAGCU CAAGC-3′; MWG Biotech, Ebersberg, Germany) was then ligated onto the 5′ ends of the TAP-treated RNA using 5 U T4 RNA Ligase (Epicentre Technologies), and the samples again cleaned by organic extraction and ethanol precipitation. The oligo-ligated RNA was then used as template for reverse transcription reactions using 200 U SuperScript II RT (Invitrogen, Carlsbad, California, United States). In each series of experiments, 20 ng each of up to 40 gene-specific primers (GSP1; sequences available on request) were used in the same reaction to generate a library of cDNAs corresponding to the mRNAs of up to 40 putative σ^E^-regulated genes. The production of full-length cDNAs was increased by reducing RNA 2° structure from incubating the reaction at increasingly higher temperatures: 37 °C for 1 h, 42 °C for 30 min, and 50 °C for 10 min. A dilution of the reverse-transcription reaction was then used as template for PCR amplification in the presence of a DNA primer containing a sequence complementary to the ligated RNA oligo sequence, and a second gene-specific primer (GSP2) for each gene that is closer to the promoter. A separate PCR reaction was performed with each GSP2 primer and the products visualized by 7.5% PAGE. Most of the tested genes contained multiple PCR products, suggesting multiple promoters. Thus, to identify σ^E^-dependent transcripts for each gene, PCR products were compared from cDNA generated from CAG25197 (*rpoE* ^+^) and CAG22216 (*rpoE* ^−^) cells; products present from *only* the *rpoE* ^+^ reactions were considered σ^E^-dependent transcripts. These products were gel-purified from 7.5% PAGE gels, electroeluted, and sequenced using the appropriate GSP2 primer. The transcription start site was defined as the nucleotide immediately preceding the sequence corresponding to the ligated RNA oligo sequence. In some cases, two adjacent start sites could be discerned by the appearance of a second RNA oligo sequence 1 nt out of frame from the first after reading the genome sequence.

### Identifying σ^E^ promoter elements upstream of transcription starts mapped by 5′ RACE

WCONSENSUS [75] was used to identify the different conserved σ^E^ promoter elements using a method similar to [6]. We note that BioOptimizer is also a suitable alternative since it can identify two-block motifs separated by a variable spacer [76]. WCONSENSUS generates optimal matrices of aligned sequence motifs based on maximizing information content and minimizing the expected frequency of finding the matrix by chance given the known sequences. Matrices were selected using the second cycle in which every sequence contributes to the final alignment. A range of sequence windows of different widths were searched to identify optimal matrices describing −10 and −35, start site, and upstream elements. Optimal matrices for the −10 motif were identified by searching sequence windows −1 to −16, and for the −35 by searching a 16-nt window 9 nt upstream of the identified −10 motif.

### σ^E^ promoter predictions using PWMs

The information content *(I_seq_)* of aligned σ^E^ promoter motifs was calculated using:



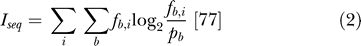



[Bibr pbio-0040002-b077]

where *i* is the position within the site, *b* refers to each of the possible bases, *f_b,i_* is the observed frequency of each base at that position, and *p_b_* is the frequency of base *b* in the entire genome (in *E. coli* taken to be 0.25 for A/G/C/T). The aligned σ^E^ promoter sequences were visualized using sequence logo ([78]; http://weblogo.berkeley.edu/).

PWMs *(W_b,i_)* for each of the σ^E^ promoter elements (PWM_UP_, PWM_−35_, PWM_−10_, and PWM_+1_) were built using the method of [79]:



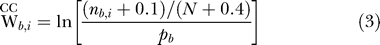



where *n_b,i_* is the number of bases *b* at position *i* in the aligned sequences and *N* is the total number of aligned sequences. A pseudo count of 0.1 was added for each base *b* for the Bayesian estimate. The relative binding affinity of σ^E^ to a DNA sequence of length *L* (equal to the length of the PWM) is given by the score:



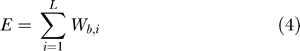



(where *b* corresponds to the nucleotide at position *i* within the sequence fragment of length *L*), such that a high score corresponds to a high-affinity site with a close match to the consensus sequence, while a low score corresponds to a low-affinity site with a poor match to the consensus. The PWM was calibrated by scoring all the sequences used to build the matrix *(E_w_),* and the distribution of the scores is described by their mean *(u_w_)* and standard deviation (*σ_w_*). Potential σ^E^ target sites in the *E. coli* genome were identified by calculating the score *E_g_* of every possible sequence window of length *L* in both strands of the genomic sequence and computing the mean *(u_g_)* and standard deviation (*σ_g_*) of the distribution. Predicted sites were made by selecting all genomic scores *E_g_* greater than a cutoff, *S_0_,* of two standard deviations below the mean of the PWM scores (*u_w_ –* 2*σ_w_*).

A penalty score adapted from the methods of [5] and [7] was applied to predicted promoters for suboptimal spacing between the +1, −10, and −35 motifs based on the observed spacing frequency for the known σ^E^ promoters. The spacer penalty was determined by taking the natural logarithm of an approximated spacer frequency normalized by the approximated frequency of the most frequently occurring spacer class. For each promoter, this was calculated for three spacers and summed to give a total spacer penalty: +1 to −10 (discriminator); −10 to −35 (spacer); and +1 to −35 (total).

A total score was calculated for each predicted promoter (*S_p_*):



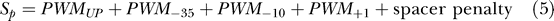



The predicted promoter scores, *S_p_,* were calibrated by scoring the known promoter sequences used to build the matrices *(S_k_)* to derive a distribution with mean *(μ_k_)* and standard deviation (*σ_k_*). The *S_p_* scores were then converted to a promoter z-score: *Z_p_* = (*S_p_* – *μ_k_*)/*σ_k_*.

### In vitro transcription assays

Single-round in vitro transcription assays were employed to test predicted σ^E^ promoters. DNA templates were prepared by PCR from genomic DNA (primer sequences available on request) to create fragments with the promoter of interest contained within flanking sequences 100 nt downstream and 200 nt upstream of the predicted transcription start point. RNA polymerase core enzyme was purified as described in [80], and His_6_-tagged σ^E^ was purified using a Qiagen Ni^2+^ affinity column per manufacturer's instructions (Valencia, California, United States). The transcription assays were performed as described in [81] with the following modifications: Binding reactions (12 μl) contained 50 nM template DNA, 250 nM core RNA polymerase, 500 nM σ^E^, 5% glycerol, 20 mM Tris (pH 8.0), 300 mM KAc, 5 mM MgAc, 0.1 mM EDTA, 1 mM DTT, 50 μg/ml BSA, and 0.05% Tween. Single-round transcriptions were initiated with 4 μl of “NTP + heparin mix” (to give a final concentration of 200 μM each NTP and 100 μg/ml heparin in 1× binding buffer), incubated for 5 min at 37 °C, and then terminated with 8 μl of 25 mM EDTA. The reactions were extracted with phenol and chloroform, precipitated with ethanol, and resuspended in 8 μl of H_2_O. The RNA transcripts were then used as templates in labeled reverse-transcription reactions using a primer ∼100 nt downstream of the predicted transcription start point (same as the downstream PCR primer used to create the template DNA). Primers were annealed by incubating with the template for 10 min at 70 °C before chilling on ice. The reverse transcription reactions (15 μl) contained 8 μl of template RNA, 10 μM primer, 1× StrataScript RT Buffer, 50 U StrataScript RNase H-RT (Stratagene, La Jolla, California, United States), 200 μM dCTP/dGTP/dTTP, 10 μM dATP, 6 μCi [α-^32^P] dATP (3,000 Ci/mmol; 110 TBq/mmol), and 8 U RNase Inhibitor (Boehringer Mannheim, Mannheim, Germany). Reactions were incubated at room temperature for 10 min and then at 42 °C for 1 h 50 min, before terminating with 9 μl of stop solution (95% deionized formamide, 25 mM EDTA, 0.05% [w/v] bromophenol blue, and 0.05% [w/v] xylene cyanol FF). The cDNA transcripts were resolved by electrophoresis after heating at 90 °C for 2 min and loading 8 μl on a 6% denaturing polyacrylamide sequencing gel together with DNA sequencing reactions that functioned as size markers. Transcripts were visualized using a Molecular Dynamics Storm 560 Phosphorimager scanning system (Sunnyvale, California, United States).

### In vivo promoter assays

Promoters to be validated were cloned on XhoI-BamHI fragments into the green fluorescent protein (GFP) reporter plasmid, pUA66 (Table 7; [82]) upstream of the gene *GFPmut2* [83]. The promoter fragments were generated by PCR from genomic DNA in which the upstream and downstream primers contained an XhoI and BamHI site, respectively, and amplified genomic promoter sequence from −65 to +20 with respect to the predicted transcription start point. Cloned promoter constructs were confirmed by sequencing. Reporter strains were generated by transforming the plasmids constructs into strains CAG25196 and CAG25197 carrying the p*Trc*99a vector and the *rpoE* expression plasmid, pLC245, respectively (Table 7). Promoter assays were performed by direct inoculation of Luria broth supplemented with appropriate antibiotics from frozen glycerol stocks. One hundred fifty–microliter cultures were grown in covered 96-well U-bottom tissue culture plates overnight at 30 °C with shaking at 400 rpm. The cultures were then diluted 1:50 into fresh 96-well plates containing Luria broth supplemented with appropriate antibiotics and 1 mM IPTG. Cultures were grown as before for up to 23 h and fluorescence measured in a Spectra Max Gemini XS 96-well fluorometer and OD_600_ measured in a Spectra Max 340 96-well spectrophotometer (Molecular Devices, Sunnyvale, California, United States). σ^E^-dependent promoter activity was determined by first subtracting the background fluorescence/OD_600_ readings of CAG25196 and CAG25197 cells bearing a promoterless GFP vector from the readings of CAG25196 and CAG25197 cells carrying the same promoter construct, and then subtracting the CAG25196 from the CAG25197 readings for each promoter. Four independent assays were performed for each promoter construct. A promoter was judged to be σ^E^ dependent if the standard deviation of the four assays did not overlap with those of the promoterless GFP vector; this translated to a σ^E^-dependent signal at least three times greater than background. This approach was validated by confirming σ^E^-dependent activity of 42 of 49 verified *E. coli* K-12 σ^E^ promoters.

### σ^E^ promoter predictions in related genomes

Promoter predictions were made in genomes as described for *E. coli* K-12 using genome sequence files (*.fna) and annotation files (*.ptt) downloaded from the NCBI FTP database (ftp://ftp://ftp.ncbi.nih.gov/genomes/Bacteria/) on 6 August 2004. For each genome promoter predictions were plotted as a function of promoter z-score versus distance upstream of the nearest ORF in the same direction (see Figure 3A). A topographic plot of promoter z-score versus distance upstream was then constructed in which the *x* and *y* axes were divided into 200-nt and 1 unit *bins*, respectively, and the number of predictions falling within each *bin (P_A_)* determined (see Figure 3B). Significant predictions were identified by comparing against predictions made in genomes containing randomized sequences. Randomized genomes were constructed to mimic the structures of real genomes but in which the nucleotide sequence of each structure was randomized. For each genome, the percentage nucleotide content was determined for all divergent IGs, convergent IGs, IGs less than 50 nt in the same direction as adjacent ORFs (short IGs), and IGs greater than 50 nt in the same direction as adjacent ORFs (long IGs). Finally, for each genome the average codon usage was determined for all ORFs. Randomized genomes of identical sizes were then constructed in which the size, orientation, and location of all the genomic structures were maintained but in which the nucleotide sequences were randomized while maintaining the average codon usage for all ORFs and the average nucleotide content for all dIGs, cIGs, long IGs, and short IGs. For each genome, promoter predictions were made from 100 randomized genomes, and, using the same bins as for the actual genomes, an averaged topographic plot was constructed that recorded the average number of predictions within each *bin* (P¯_R_; see Figure 3C). For each bin of the actual genome topographic plot, a FPR was calculated that compared the average number of predictions in the 100 randomized genomes (P¯_R_) with the number of predictions in the actual genome *(P_A_):*



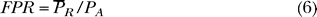



In addition, for each *bin*, the significance of obtaining the observed number of predictions from the actual genome *(P_A_)* given the average number of prediction from the randomized genomes (P¯_R_) was calculated based on Poisson distribution to derive a *p*-value. All promoter predictions in actual genomes were assigned a FPR and *p*-value based on the *bin* where they were located. Promoter predictions for an actual genome were determined significant if, in general, FPR < 0.5 and *p* < 0.05, with the FPR cutoff being the stricter filter. Additional filters of promoter z-score > −2, distance upstream <1,100 nt were also applied to prevent spurious results in some genomes.

### Conserved σ^E^ promoter predictions

A database of protein orthologs across the genomes was constructed using the program BLAST and the NCBI protein sequence files (*.faa) for each genome. Orthologs were defined as the highest scoring hit in a target genome, which, when the matching sequence was used to search the original genome, identified the same search sequence as the highest scoring match. All coding sequences in the genomes were organized into putative TUs defined as all adjacent ORFs in the same orientation separated by less than 50 nt [84]. Using the protein ortholog database, conserved TUs across genomes were identified by containing at least one protein ortholog. In some instances, a TU in one genome may match more than one TU in other genomes due to the location of constituent ORFs becoming separated. Conserved promoter predictions were defined as predictions from the promoter prediction libraries less than 1,100 nt upstream of all orthologous TUs and scored in general promoter z-score > −2, distances upstream < −1,100 nt, FPR <0.5, and *p* < 0.05 in at least one genome. Given that each promoter library contains approximately 150 predictions with z-score > −2 at distances <1,100 nt upstream, and each genome contains on average 4,500 genes, a matching promoter occurring by random chance for a particular search promoter = 150 of 4,500, or 0.033.

## Supporting Information

Figure S1 Amino Acid Sequence Alignments of Conserved DNA-Binding Regions of σ^E^ across Eight Genomes The RpoE (σ^E^) sequences are aligned against RpoD (σ^70^) based on the structural alignment in [30]. Residues inferred to be involved in DNA interactions are based from σ^70^ [31] and are highlighted in yellow.
(A) Alignments of conserved regions 2.2–3.0 involved in −10 promoter recognition.
(B) Alignments of conserved regions 4.1–4.2 involved in −35 promoter recognition.
K-12, *E. coli* K-12; CFT073, *E. coli* CFT073; O157, *E. coli* O157:H7 EDL933; Sfl, *Shigella flexneri 2a* str. 2457T; Sty, *Salmonella enterica* subsp*. enterica* serovar Typhi str. CT18; Stm, *Salmonella typhimurium LT2;* Plu, *Photorhabdus luminescens* subsp*. laumondii* TTO1; Eca, *Erwinia carotovora* subsp*. atroseptica* SCR11043; Ype, *Yersinia pestis* CO92.
(64 KB PDF).

Table S1 Highly Significant and Conserved σ^E^ Promoter Predictions across Nine Closely Related Genomes Orthologous TUs are displayed on the same row; note that only one gene in each TU needs to be an ortholog. Genes within a TU are separated by “=” in the following fields: Unique ID (unique identification number from NCBI ptt file); Gene (Gene name); Function (Gene description from NCBI ptt file). Promoter predictions are given in the fields Distance (number of nucleotides of +1 position upstream of translation start point of the first gene in the TU) and Score (total promoter z-score; see Materials and Methods). If there is no promoter prediction for that TU, these two fields just contain “–.” Promoter predictions for *E. coli* K-12, *E. coli* CFT073, and *S. typhimurium* highlighted in gray in the distance and score fields have been validated by in vitro transcriptions and/or in vivo promoter assays. Promoter predictions in *E. coli* CFT073 that are conserved with *E. coli* K-12 are presumed functional based on their high level of conservation and were not tested. See Figure S1 for abbreviations.
(100 KB XLS).

Table S2 σ^E^ Regulon Members in Nine Closely Related Genomes Organized into the Functional Categories Displayed in Table 5
Orthologous proteins are displayed on the same row. Proteins in parenthesis are part of TUs observed to be regulated in *E. coli* K-12 and based on TU conservation are assumed to be part of the regulon in the related genomes. Validated predictions for *E. coli* K-12, *E. coli* CFT073, and *S. typhimurium* are highlighted in gray. Predictions in *E. coli* CFT073 that are conserved with *E. coli* K-12 are presumed functional based on their high level of conservation and were not tested. See Figure S1 for abbreviations.
(27 KB XLS).

### Accession Numbers

The National Center for Biotechnology (NCBI) (http://www.ncbi.nlm.nih.gov/) accession numbers for the bacteria discussed in this paper are *Erwinia carotovora* subsp. *atroseptica* SCRI1043 (NC_004547); *E. coli* K-12 MG1655 (NC_000913); *E. coli* CFT073 (NC_004431); *E. coli* O157:H7 EDL933 (NC_002655); *Photorhabdus luminescens* subsp. *laumondii* TTO1 (NC_005126); *Salmonella enterica* subsp. *enterica* serovar Typhi str. CT18 (NC_003198); *Salmonella typhimurium* LT2 (NC_003197); *Shigella flexneri 2a* str. 2457T (NC_004741); and *Yersinia pestis* CO92 (NC_003143). Raw and normalized microarray expression data are available on the NCBI GEO Web site (http://www.ncbi.nlm.nih.gov/geo/) under the accession code GSE3437.

## Abbreviations

5′ RACE: rapid amplification of cDNA ends
FPR: false-positive rate
GFP: green fluorescent protein
IG: intergenic region
LPS: lipopolysaccharide
O-PS: outer polysaccharide
OMP: outer membrane porin
ORF: open reading frame
PWM: position weight matrix
RNAP: RNA polymerase
SAM: statistical analysis of microarrays
TU: transcription unit
UP: upstream element

## References

[pbio-0040002-b001] Demeler B, Zhou GW (1991). Neural network optimization for *E. coli* promoter prediction. Nucleic Acids Res.

[pbio-0040002-b002] Burden S, Lin YX, Zhang R (2004). Improving promoter prediction for the NNPP2.2 algorithm: A case study using *Escherichia coli* DNA sequences. Bioinformatics.

[pbio-0040002-b003] Horton PB, Kanehisa M (1992). An assessment of neural network and statistical approaches for prediction of *E. coli* promoter sites. Nucleic Acids Res.

[pbio-0040002-b004] O'Neill MC (1992). *Escherichia coli* promoters: Neural networks develop distinct descriptions in learning to search for promoters of different spacing classes. Nucleic Acids Res.

[pbio-0040002-b005] Staden R (1984). Computer methods to locate signals in nucleic acid sequences. Nucleic Acids Res.

[pbio-0040002-b006] Huerta AM, Collado-Vides J (2003). Sigma70 promoters in *Escherichia coli*: Specific transcription in dense regions of overlapping promoter-like signals. J Mol Biol.

[pbio-0040002-b007] Hertz GZ, Stormo GD (1996). *Escherichia coli* promoter sequences: Analysis and prediction. Methods Enzymol.

[pbio-0040002-b008] Mulligan ME, Hawley DK, Entriken R, McClure WR (1984). *Escherichia coli* promoter sequences predict in vitro RNA polymerase selectivity. Nucleic Acids Res.

[pbio-0040002-b009] Cao M, Kobel PA, Morshedi MM, Wu MF, Paddon C (2002). Defining the *Bacillus subtilis* sigma(W) regulon: A comparative analysis of promoter consensus search, run-off transcription/macroarray analysis (ROMA), and transcriptional profiling approaches. J Mol Biol.

[pbio-0040002-b010] Gruber TM, Gross CA (2003). Multiple sigma subunits and the partitioning of bacterial transcription space. Annu Rev Microbiol.

[pbio-0040002-b011] Helmann JD (2002). The extracytoplasmic function (ECF) sigma factors. Adv Microb Physiol.

[pbio-0040002-b012] Alba BM, Gross CA (2004). Regulation of the *Escherichia coli* sigma-dependent envelope stress response. Mol Microbiol.

[pbio-0040002-b013] Raivio TL, Silhavy TJ (2001). Periplasmic stress and ECF sigma factors. Annu Rev Microbiol.

[pbio-0040002-b014] De Las Penas A, Connolly L, Gross CA (1997). SigmaE is an essential sigma factor in *Escherichia coli*. J Bacteriol.

[pbio-0040002-b015] Humphreys S, Stevenson A, Bacon A, Weinhardt AB, Roberts M (1999). The alternative sigma factor, sigmaE, is critically important for the virulence of *Salmonella typhimurium*. Infect Immun.

[pbio-0040002-b016] Redford P, Roesch PL, Welch RA (2003). DegS is necessary for virulence and is among extraintestinal *Escherichia coli* genes induced in murine peritonitis. Infect Immun.

[pbio-0040002-b017] Testerman TL, Vazquez-Torres A, Xu Y, Jones-Carson J, Libby SJ (2002). The alternative sigma factor sigmaE controls antioxidant defences required for *Salmonella* virulence and stationary-phase survival. Mol Microbiol.

[pbio-0040002-b018] Craig JE, Nobbs A, High NJ (2002). The extracytoplasmic sigma factor, final sigma(E), is required for intracellular survival of nontypeable *Haemophilus influenzae* in J774 macrophages. Infect Immun.

[pbio-0040002-b019] Kovacikova G, Skorupski K (2002). The alternative sigma factor sigma(E) plays an important role in intestinal survival and virulence in *Vibrio cholerae*. Infect Immun.

[pbio-0040002-b020] Martin DW, Schurr MJ, Yu H, Deretic V (1994). Analysis of promoters controlled by the putative sigma factor AlgU regulating conversion to mucoidy in *Pseudomonas aeruginosa*: Relationship to sigma E and stress response. J Bacteriol.

[pbio-0040002-b021] Yu H, Schurr MJ, Deretic V (1995). Functional equivalence of *Escherichia coli* sigma E and *Pseudomonas aeruginosa* AlgU: *E. coli* rpoE restores mucoidy and reduces sensitivity to reactive oxygen intermediates in algU mutants of *P. aeruginosa*. J Bacteriol.

[pbio-0040002-b022] Humphreys S, Rowley G, Stevenson A, Kenyon WJ, Spector MP (2003). Role of periplasmic peptidylprolyl isomerases in *Salmonella enterica* serovar Typhimurium virulence. Infect Immun.

[pbio-0040002-b023] Gonzalez AD, Espinosa V, Vasconcelos AT, Perez-Rueda E, Collado-Vides J (2005). TRACTOR_DB: A database of regulatory networks in gamma-proteobacterial genomes. Nucleic Acids Res.

[pbio-0040002-b024] Tan K, Moreno-Hagelsieb G, Collado-Vides J, Stormo GD (2001). A comparative genomics approach to prediction of new members of regulons. Genome Res.

[pbio-0040002-b025] Erill I, Escribano M, Campoy S, Barbe J (2003). In silico analysis reveals substantial variability in the gene contents of the gamma proteobacteria LexA-regulon. Bioinformatics.

[pbio-0040002-b026] Tusher VG, Tibshirani R, Chu G (2001). Significance analysis of microarrays applied to the ionizing radiation response. Proc Natl Acad Sci U S A.

[pbio-0040002-b027] Estrem ST, Gaal T, Ross W, Gourse RL (1998). Identification of an UP element consensus sequence for bacterial promoters. Proc Natl Acad Sci U S A.

[pbio-0040002-b028] Dartigalongue C, Missiakas D, Raina S (2001). Characterization of the *Escherichia coli* sigma E regulon. J Biol Chem.

[pbio-0040002-b029] Rezuchova B, Miticka H, Homerova D, Roberts M, Kormanec J (2003). New members of the *Escherichia coli* sigmaE regulon identified by a two-plasmid system. FEMS Microbiol Lett.

[pbio-0040002-b030] Campbell EA, Tupy JL, Gruber TM, Wang S, Sharp MM (2003). Crystal structure of *Escherichia coli* sigmaE with the cytoplasmic domain of its anti-sigma RseA. Mol Cell.

[pbio-0040002-b031] Campbell EA, Muzzin O, Chlenov M, Sun JL, Olson CA (2002). Structure of the bacterial RNA polymerase promoter specificity sigma subunit. Mol Cell.

[pbio-0040002-b032] Moreno-Hagelsieb G, Collado-Vides J (2002). A powerful non-homology method for the prediction of operons in prokaryotes. Bioinformatics.

[pbio-0040002-b033] Firoved AM, Boucher JC, Deretic V (2002). Global genomic analysis of AlgU (sigma(E))-dependent promoters (sigmulon) in *Pseudomonas aeruginosa* and implications for inflammatory processes in cystic fibrosis. J Bacteriol.

[pbio-0040002-b034] Cases I, Ussery DW, de Lorenzo V (2003). The sigma54 regulon (sigmulon) of *Pseudomonas putida*. Environ Microbiol.

[pbio-0040002-b035] Reitzer L, Schneider BL (2001). Metabolic context and possible physiological themes of sigma(54)-dependent genes in *Escherichia coli*. Microbiol Mol Biol Rev.

[pbio-0040002-b036] Dombrecht B, Marchal K, Vanderleyden J, Michiels J (2002). Prediction and overview of the RpoN-regulon in closely related species of the Rhizobiales. Genome Biol.

[pbio-0040002-b037] Nikaido H, Neidhardt FC, Curtiss R, Ingraham JL, Lin ECC, Low KB (1996). Outer membrane. *Escherichia coli* and *Salmonella*: Cellular and molecular miology.

[pbio-0040002-b038] Raetz CR, Whitfield C (2002). Lipopolysaccharide endotoxins. Annu Rev Biochem.

[pbio-0040002-b039] El Ghachi M, Bouhss A, Blanot D, Mengin-Lecreulx D (2004). The bacA gene of *Escherichia coli* encodes an undecaprenyl pyrophosphate phosphatase activity. J Biol Chem.

[pbio-0040002-b040] Nikaido H (2003). Molecular basis of bacterial outer membrane permeability revisited. Microbiol Mol Biol Rev.

[pbio-0040002-b041] Onufryk C, Crouch ML, Fang FC, Gross CA (2005). Characterization of six lipoproteins in the sigmaE regulon. J Bacteriol.

[pbio-0040002-b042] Wu T, Malinverni J, Ruiz N, Kim S, Silhavy TJ (2005). Identification of a multicomponent complex required for outer membrane biogenesis in *Escherichia coli*. Cell.

[pbio-0040002-b043] Rizzitello AE, Harper JR, Silhavy TJ (2001). Genetic evidence for parallel pathways of chaperone activity in the periplasm of *Escherichia coli*. J Bacteriol.

[pbio-0040002-b044] Voulhoux R, Tommassen J (2004). Omp85, an evolutionarily conserved bacterial protein involved in outer-membrane-protein assembly. Res Microbiol.

[pbio-0040002-b045] Gentle I, Gabriel K, Beech P, Waller R, Lithgow T (2004). The Omp85 family of proteins is essential for outer membrane biogenesis in mitochondria and bacteria. J Cell Biol.

[pbio-0040002-b046] Paschen SA, Waizenegger T, Stan T, Preuss M, Cyrklaff M (2003). Evolutionary conservation of biogenesis of beta-barrel membrane proteins. Nature.

[pbio-0040002-b047] Braun M, Silhavy TJ (2002). Imp/OstA is required for cell envelope biogenesis in *Escherichia coli*. Mol Microbiol.

[pbio-0040002-b048] Kloser A, Laird M, Deng M, Misra R (1998). Modulations in lipid A and phospholipid biosynthesis pathways influence outer membrane protein assembly in *Escherichia coli* K-12. Mol Microbiol.

[pbio-0040002-b049] Ried G, Hindennach I, Henning U (1990). Role of lipopolysaccharide in assembly of *Escherichia coli* outer membrane proteins OmpA, OmpC, and OmpF. J Bacteriol.

[pbio-0040002-b050] Bos MP, Tefsen B, Geurtsen J, Tommassen J (2004). Identification of an outer membrane protein required for the transport of lipopolysaccharide to the bacterial cell surface. Proc Natl Acad Sci U S A.

[pbio-0040002-b051] Pages JM, Bolla JM, Bernadac A, Fourel D (1990). Immunological approach of assembly and topology of OmpF, an outer membrane protein of *Escherichia coli*. Biochimie.

[pbio-0040002-b052] de Cock H, Pasveer M, Tommassen J, Bouveret E (2001). Identification of phospholipids as new components that assist in the in vitro trimerization of a bacterial pore protein. Eur J Biochem.

[pbio-0040002-b053] Janakiraman A, Goldberg MB (2004). Recent advances on the development of bacterial poles. Trends Microbiol.

[pbio-0040002-b054] Walsh NP, Alba BM, Bose B, Gross CA, Sauer RT (2003). OMP peptide signals initiate the envelope-stress response by activating DegS protease via relief of inhibition mediated by its PDZ domain. Cell.

[pbio-0040002-b055] Ades SE, Grigorova IL, Gross CA (2003). Regulation of the alternative sigma factor sigma(E) during initiation, adaptation, and shutoff of the extracytoplasmic heat shock response in *Escherichia coli*. J Bacteriol.

[pbio-0040002-b056] Ades SE, Connolly LE, Alba BM, Gross CA (1999). The *Escherichia coli* sigma(E)-dependent extracytoplasmic stress response is controlled by the regulated proteolysis of an anti-sigma factor. Genes Dev.

[pbio-0040002-b057] Alba BM, Leeds JA, Onufryk C, Lu CZ, Gross CA (2002). DegS and YaeL participate sequentially in the cleavage of RseA to activate the sigma(E)-dependent extracytoplasmic stress response. Genes Dev.

[pbio-0040002-b058] Alba BM, Zhong HJ, Pelayo JC, Gross CA (2001). degS (hhoB) is an essential *Escherichia coli* gene whose indispensable function is to provide sigma activity. Mol Microbiol.

[pbio-0040002-b059] Kanehara K, Ito K, Akiyama Y (2002). YaeL (EcfE) activates the sigma(E) pathway of stress response through a site-2 cleavage of anti-sigma(E), RseA. Genes Dev.

[pbio-0040002-b060] Grigorova IL, Chaba R, Zhong HJ, Alba BM, Rhodius V (2004). Fine-tuning of the *Escherichia coli* sigmaE envelope stress response relies on multiple mechanisms to inhibit signal-independent proteolysis of the transmembrane anti-sigma factor, RseA. Genes Dev.

[pbio-0040002-b061] Wren BW (2000). Microbial genome analysis: insights into virulence, host adaptation and evolution. Nat Rev Genet.

[pbio-0040002-b062] Rahme LG, Ausubel FM, Cao H, Drenkard E, Goumnerov BC (2000). Plants and animals share functionally common bacterial virulence factors. Proc Natl Acad Sci U S A.

[pbio-0040002-b063] Finlay BB, Falkow S (1997). Common themes in microbial pathogenicity revisited. Microbiol Mol Biol Rev.

[pbio-0040002-b064] Sambrook J, Fritsch EF, Maniatis T (1989). Molecular cloning: A laboratory manual.

[pbio-0040002-b065] Arber W, Enquist L, Hohn B, Murray NE, Murray K, Hendrix RW, Roberts JW, Stahl FW, Weisberg RA (1983). Experimental methods for use with lambda. Lambda II.

[pbio-0040002-b066] Miller JH (1992). A short course in bacterial genetics: A laboratory manual and handbook for *Escherichia coli* and related bacteria.

[pbio-0040002-b067] Tao H, Bausch C, Richmond C, Blattner FR, Conway T (1999). Functional genomics: Expression analysis of *Escherichia coli* growing on minimal and rich media. J Bacteriol.

[pbio-0040002-b068] Khodursky AB, Bernstein JA, Peter BJ, Rhodius V, Wendisch VF (2003). *Escherichia coli* spotted double-strand DNA microarrays: RNA extraction, labeling, hybridization, quality control, and data management. Methods Mol Biol.

[pbio-0040002-b069] Schena M, Shalon D, Davis RW, Brown PO (1995). Quantitative monitoring of gene expression patterns with a complementary DNA microarray. Science.

[pbio-0040002-b070] Richmond CS, Glasner JD, Mau R, Jin H, Blattner FR (1999). Genome-wide expression profiling in *Escherichia coli* K-12. Nucleic Acids Res.

[pbio-0040002-b071] Rhodius V, Van Dyk TK, Gross C, LaRossa RA (2002). Impact of genomic technologies on studies of bacterial gene expression. Annu Rev Microbiol.

[pbio-0040002-b072] Tseng GC, Oh MK, Rohlin L, Liao JC, Wong WH (2001). Issues in cDNA microarray analysis: Quality filtering, channel normalization, models of variations and assessment of gene effects. Nucleic Acids Res.

[pbio-0040002-b073] Yang YH, Dudoit S, Luu P, Lin DM, Peng V (2002). Normalization for cDNA microarray data: A robust composite method addressing single and multiple slide systematic variation. Nucleic Acids Res.

[pbio-0040002-b074] Frohman MA (1994). On beyond classic RACE (rapid amplification of cDNA ends). PCR Methods Appl.

[pbio-0040002-b075] Hertz GZ, Stormo GD (1999). Identifying DNA and protein patterns with statistically significant alignments of multiple sequences. Bioinformatics.

[pbio-0040002-b076] Jensen ST, Liu JS (2004). BioOptimizer: A Bayesian scoring function approach to motif discovery. Bioinformatics.

[pbio-0040002-b077] Schneider TD, Stormo GD, Gold L, Ehrenfeucht A (1986). Information content of binding sites on nucleotide sequences. J Mol Biol.

[pbio-0040002-b078] Crooks GE, Hon G, Chandonia JM, Brenner SE (2004). WebLogo: A sequence logo generator. Genome Res.

[pbio-0040002-b079] Stormo GD (1990). Consensus patterns in DNA. Methods Enzymol.

[pbio-0040002-b080] Young BA, Anthony LC, Gruber TM, Arthur TM, Heyduk E (2001). A coiled-coil from the RNA polymerase beta' subunit allosterically induces selective nontemplate strand binding by sigma(70). Cell.

[pbio-0040002-b081] Rhodius V, Savery N, Kolb A, Busby S (2001). Assays for transcription factor activity. Methods Mol Biol.

[pbio-0040002-b082] Zaslaver A, Mayo AE, Rosenberg R, Bashkin P, Sberro H (2004). Just-in-time transcription program in metabolic pathways. Nat Genet.

[pbio-0040002-b083] Cormack BP, Valdivia RH, Falkow S (1996). FACS-optimized mutants of the green fluorescent protein (GFP). Gene.

[pbio-0040002-b084] Salgado H, Moreno-Hagelsieb G, Smith TF, Collado-Vides J (2000). Operons in *Escherichia coli*: Genomic analyses and predictions. Proc Natl Acad Sci U S A.

[pbio-0040002-b085] Flynn JM, Levchenko I, Sauer RT, Baker TA (2004). Modulating substrate choice: The SspB adaptor delivers a regulator of the extracytoplasmic-stress response to the AAA+ protease ClpXP for degradation. Genes Dev.

[pbio-0040002-b086] Lipinska B, Sharma S, Georgopoulos C (1988). Sequence analysis and regulation of the htrA gene of *Escherichia coli*: A sigma 32-independent mechanism of heat-inducible transcription. Nucleic Acids Res.

[pbio-0040002-b087] Erickson JW, Gross CA (1989). Identification of the sigma E subunit of *Escherichia coli* RNA polymerase: A second alternate sigma factor involved in high-temperature gene expression. Genes Dev.

[pbio-0040002-b088] Rouviere PE, De Las Penas A, Mecsas J, Lu CZ, Rudd KE (1995). rpoE, the gene encoding the second heat-shock sigma factor, sigma E, in *Escherichia coli*. EMBO J.

[pbio-0040002-b089] Danese PN, Silhavy TJ (1997). The sigma(E) and the Cpx signal transduction systems control the synthesis of periplasmic protein-folding enzymes in *Escherichia coli*. Genes Dev.

[pbio-0040002-b090] Erickson JW, Vaughn V, Walter WA, Neidhardt FC, Gross CA (1987). Regulation of the promoters and transcripts of rpoH, the *Escherichia coli* heat shock regulatory gene. Genes Dev.

[pbio-0040002-b091] Casadaban MJ, Cohen SN (1980). Analysis of gene control signals by DNA fusion and cloning in *Escherichia coli*. J Mol Biol.

[pbio-0040002-b092] Guyer MS, Reed RR, Steitz JA, Low KB (1981). Identification of a sex-factor-affinity site in *E. coli* as gamma delta. Cold Spring Harb Symp Quant Biol.

[pbio-0040002-b093] Jensen KF (1993). The *Escherichia coli* K-12 “wild types” W3110 and MG1655 have an rph frameshift mutation that leads to pyrimidine starvation due to low pyrE expression levels. J Bacteriol.

[pbio-0040002-b094] Mecsas J, Rouviere PE, Erickson JW, Donohue TJ, Gross CA (1993). The activity of sigma E, an *Escherichia coli* heat-inducible sigma-factor, is modulated by expression of outer membrane proteins. Genes Dev.

