# Supplementary material for: Conserved and Variable Functions of the σE Stress Response in Related Genomes
Source: PLoS Biol. 2005 Dec 20;4(1):e2. doi: 10.1371/journal.pbio.0040002 (PMC1312014; doi:10.1371/journal.pbio.0040002)
Supplement: Supplementary file 1 [file pbio.0040002.sg001.pdf]

A

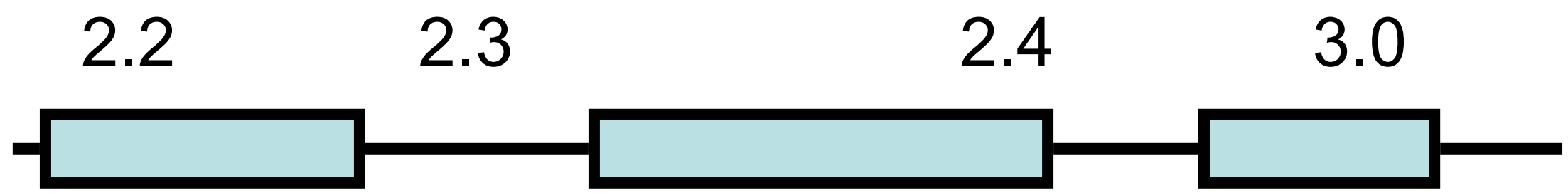


```
K-12 rpoD    403 DLIQEGNIGLMKAVDKFEYRRGYK-FSTYATWWIRQAITRSIADQARTIRIPVHMIETINKLNRISRQ---
K-12 rpoE     45 DVPDVVQEAFIKAYRALDSFRGDSAFYTWLYRIAVNTAKNYLVAQGR--RPPSSDVDAIEAENFESGG---
CFT073 rpoE   54 DVPDVVQEAFIKAYRALDSFRGDSAFYTWLYRIAVNTAKNYLVAQGR--RPPSSDVDAIEAENFESGG---
Sfl rpoE      45 DVPDVVQEAFIKAYRALDSFRGDSAFYTWLYRIAVNTAKNYLVAQGR--RPPSSDVDAIEAENFESGG---
O157 rpoE     45 DVPDVVQEAFIKAYRALDSFRGDSAFYTWLYRIAVNTAKNYLVAQGR--RPPSSDVDAIEAENFESGG---
Stm rpoE      45 DVPDVVQESFIKAYRALDSFRGDSAFYTWLYRIAVNTAKNYLVAQGR--RPPSSDVDAIEAENFESGG---
Stm rpoE      45 DVPDVVQESFIKAYRALDSFRGDSAFYTWLYRIAVNTAKNYLVAQGR--RPPSSDVDAIEAENFESGG---
Ype rpoE      45 DVPDVVQESFIKAYRALESFRGDSAFYTWLYRIAVNTAKNYLVAQGR--RPPSSDVDANDAENYENAG---
Eca rpoE      45 DVPDVVQESFIKAYRALESFRGDSAFYTWLYRIAVNTAKNYLVAQGR--RPPSSDIDANDAENYENAG---
Plu rpoE      45 DVPDVAQETFIKAYRALASFRGDSAFYTWLYRIAVNTAKNYLTAQGR--RPPSSDLDASDAENYEISS---
```

B

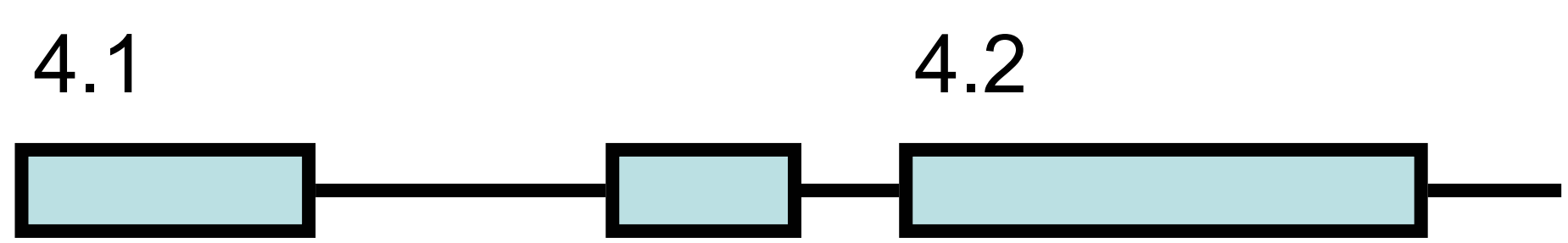


```
K-12 rpoD    553 AREAKVLRMRF--GIDMNTDYTLEEVGKQFDVTRERIRQIEAKALRKLRHPSRSEVLRSFLDD
K-12 rpoE    140 EDLRMAITLRELDGL------SYEEIAAIMDCPVGTVRSRIFRAREAIDNKVQPLIRR
CFT073 rpoE  151 EDLRMAITLRELDGL------SYEEIAAIMDCPVGTVRSRIFRAREAIDNKVQPLIRR
Sfl rpoE     140 EDLRMAITLRELDGL------SYEEIAAIMDCPVGTVRSRIFRAREAIDNKVQPLIRR
O157 rpoE    140 EDLRMAITLRELDGL------SYEEIAAIMDCPVGTVRSRIFRAREAIDNKVQPLIRR
Sty rpoE     140 EDLRMAITLRELDGL------SYEEIAAIMDCPVGTVRSRIFRAREAIDNKVQPLIRR
Stm rpoE     140 EDLRMAITLRELDGL------SYEEIAAIMDCPVGTVRSRIFRAREAIDNKVQPLIRR
Ype rpoE     140 EDLRMAITLRELDGL------SYEEIAAIMDCPVGTVRSRIFRAREAIDNKVQPLIQR
Eca rpoE     140 EDLRLAITLRELDGL------SYEEIAVIMDCPVGTVRSRIFRAREAIDNKVQPLIQR
Plu rpoE     140 EDLRIAIMLRELDGL------SYEEIATIMNCPVGTVRSRIFRAREAIDDNIQPLI
```
